# Gearing effects of the patella (knee extensor muscle sesamoid) of the helmeted guineafowl during terrestrial locomotion

**DOI:** 10.1111/jzo.12485

**Published:** 2017-07-19

**Authors:** V. R. Allen, R. E. Kambic, S. M. Gatesy, J. R. Hutchinson

**Affiliations:** ^1^ Structure & Motion Laboratory Department of Comparative Biomedical Sciences Royal Veterinary College Hatfield Hertfordshire UK; ^2^ Brown University Providence RI USA; ^3^ Harvard University Boston MA USA

**Keywords:** avian, kinematics, locomotion, biomechanics, helmeted guineafowl, patella, *Numida meleagris*, knee extensor

## Abstract

Human patellae (kneecaps) are thought to act as gears, altering the mechanical advantage of knee extensor muscles during running. Similar sesamoids have evolved in the knee extensor tendon independently in birds, but it is unknown if these also affect the mechanical advantage of knee extensors. Here, we examine the mechanics of the patellofemoral joint in the helmeted guineafowl *Numida meleagris* using a method based on muscle and tendon moment arms taken about the patella's rotation centre around the distal femur. Moment arms were estimated from a computer model representing hindlimb anatomy, using hip, knee and patellar kinematics acquired via marker‐based biplanar fluoroscopy from a subject running at 1.6 ms^−1^ on a treadmill. Our results support the inference that the patella of *Numida* does alter knee extensor leverage during running, but with a mechanical advantage generally greater than that seen in humans, implying relatively greater extension force but relatively lesser extension velocity.

## Introduction

Patella‐like bones (knee sesamoids, *sensu* Vickaryous & Olson, 2007) are found within the knee extensor tendons of most extant mammals and many birds and lizards. Yet, as with sesamoids in general, our understanding of why these bones form and what (if any) functions they perform remains limited. In human knees, tension in the common knee extensor tendon has been observed (Bishop, 1977; Ellis *et al*., 1980; Feller *et al*., 2007) to differ between the portion distal to the patella (the patellar tendon) and the portion proximal to it (the extensor muscle tendon). Mathematical modelling (Eijden *et al*., 1986) suggests that this can be explained by the patellar and extensor tendons having different leverage about the patellofemoral joint. The patellar complex (i.e. the proximal extensor tendon, distal patellar tendon and patellofemoral joint) therefore affects the mechanical advantage (output force/input force) of the knee extensors. Mechanical advantage affects not only output force but also output velocity, which will vary in inverse proportion to it (almost directly so if friction is negligible). This indicates that the human patella acts as a form of idler gear between the knee extensor muscles and the knee joint itself, allowing the force and velocity with which the muscles contract to differ from the force and velocity with which the patellar tendon pulls on its attachment to the tibia.

**Figure 1:**
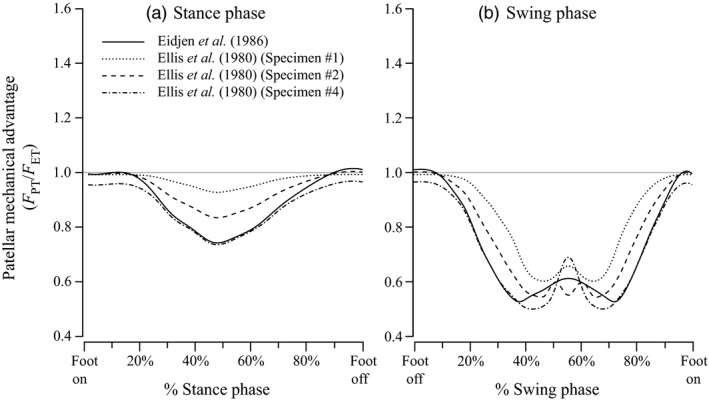
Ratios of tension in the patellar tendon to tension in the main knee extensor tendon (*F*_PT_/*F*_ET_, or patellar mechanical advantage) reported in previous studies of humans, plotted against percentage of (a) stance and (b) swing phase. Solid lines show data from Eijden *et al*. (1986), and all others show specimens from Ellis *et al*. (1980): Dotted lines show specimen #1, dashed lines show #2 and dash‐dots show specimen #4.

Tension ratios between the extensor muscle tendon and the patellar tendon suggest that the mechanical advantage of the human patellar complex is greater than 1.0 (amplifying force) only near full knee extension (Eijden *et al*., 1986). Over the remaining flexion/extension range, the mechanical advantage appears less than 1.0, indicating velocity rather than force is being amplified (Bishop, 1977; Ellis *et al*., 1980; Feller *et al*., 2007). As the knee does not extend fully during running, and only extends fully at the very beginning and end of the stance phase during walking (e.g. Biewener *et al*., 2004), this implies that the mechanical advantage of the human patellar complex is mostly less than 1.0 during locomotion, and nearly exclusively so when running (Fig. 1). Assuming the angular velocity and torque of the knee joint is proportional to the linear velocity and force with which the patellar tendon actuates it, the patella of humans seems to amplify knee extension velocity (and reduce knee extension torque) during walking and running.

This raises questions about the patellar sesamoids of other tetrapod vertebrates. Do these structures, which have evolved independently in birds, mammals and lizards (Dye, 1987; Sarin *et al*., 1999; Regnault, Pitsillides & Hutchinson, 2014; Regnault *et al*., 2016) share a similar gearing function? If so, why do some taxa have sesamoids in their knee extensor tendons, whereas others (even if similar in form and behaviour) do not? Are knee mechanics fundamentally different between taxa with patellae and taxa without? Broader knowledge of patellar mechanics could thus greatly enhance our knowledge of knee joint function in general, but comparative analysis is currently hampered by a lack of non‐human or non‐mammalian data. Here, we make an initial step in addressing this deficiency by analysing the mechanical advantage of the patellar complex in a terrestrially locomoting bird, the helmeted guineafowl *Numida meleagris* Linnaeus 1758.

## Materials and methods

### Sesamoid biomechanics: theory

Direct measurement of mechanical advantage (output force/input force) requires implantation of tension‐sensing equipment (e.g. tendon buckles) into the knee. This makes its *in vivo* application to walking/running taxa problematic, so such studies are restricted to cadaveric material. Patellar mechanical advantage has also been estimated using mathematical models based on bone positions and muscle–tendon lines of action taken from radiographs of the patellar complex, both *ex vivo* by Eijden *et al*. (1986) and *in vivo* by Alexander & Dimery (1985).

We base our method on the latter study. We assume that the forces and movements of the patellar complex during locomotion are restricted to a single plane. We assume that conditions equivalent to static equilibrium apply. All joints are assumed to be frictionless and unimpeded in their normal motion by tension in the collateral ligaments, and forces due to limb segment mass (weight and inertia) are assumed negligible. The only appreciable forces involved are therefore assumed to be those exerted by the muscle–tendon units, those due to the weight of the animal, and those due to contact between the bones of the joints.

**Figure 2:**
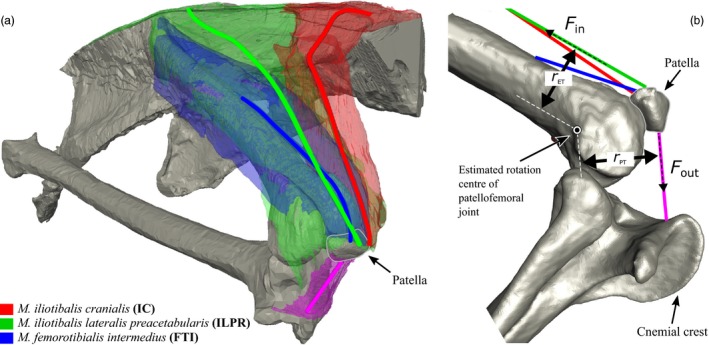
(a) Anatomy of knee extensor muscles attaching to the patella in Numida. Solid lines represent lines of action for each muscle, estimated as the centre of cross‐sections taken from anatomical origin to insertion. The *M. iliotibialis cranialis* is shown in red, *M. iliotibialis lateralis preacetabularis* in green and *M. femorotibialis intermedius* in blue. (b) Schematic of input data for Equation (1): *r*_ET_ and *r*_PT_ are the moment arms of the knee extensor tendon(s) and patellar tendon, respectively, about the estimated rotation centre of the patella as it orbits the knee. F_in_ and F_out_ are the ‘input’ and ‘output’ tensions in the knee extensor tendon(s) and the patellar tendon, respectively.

Given these conditions, at any time the proximal knee extensor tendon and the distal patellar tendon exert equal‐and‐opposite torques about the instantaneous rotation centre of the patellofemoral joint (Fig. 2b). The mechanical advantage can then be calculated using the moment arm of each tendon about the instant centre, as described by equations (1) and (2) (below):

(1) $$F_{\mathrm{PT}}=\frac{F_{\mathrm{ET}}r_{\mathrm{ET}}}{r_{PT}}$$

(2) $$V_{\mathrm{PT}}=\frac{V_{\mathrm{ET}}r_{\mathrm{PT}}}{r_{ET}}$$

where *F*_PT_, *V*_PT_ and *r*_PT_ are the tension, velocity and moment arm, respectively, of the patellar tendon, and *F*_ET_, *V*_ET_ and *r*_ET_ are the same for the knee extensor tendon (see Fig. 2b). The fraction *r*_ET_/*r*_PT_ represents the mechanical advantage of the system, and its inverse the ratio of output to input velocity (if friction is negligible). Again, if knee joint angular velocity and torque is proportional to the linear velocity and force with which the patellar tendon actuates it, for each extensor muscle in the patellar complex, the difference between contractile force and velocity and the resulting knee extension force and velocity should be proportional to the mechanical advantage.

### Specimen

We used kinematic data collected during a previous study (Kambic, Roberts & Gatesy, 2015). Only one specimen used in Kambic *et al*. (2015), an adult (1.41 kg) female, was implanted with a patella marker, and so we are limited to data from this individual. While this is not ideal, the *Numida* used here is reasonably representative, with a body mass within one standard deviation of the species mean (1.29 ± 0.19 kg, Nalubamba, Mudenda & Masuku, 2010), and kinematic data for the hip and knee are in good agreement with previously published datasets on fast‐moving *Numida* (Gatesy, 1999a; Kambic *et al*., 2015; see Fig. S1).

### Skeletal Kinematics

We briefly recount methodology from Kambic *et al*. (2015) here. Skeletal kinematics was estimated using marker‐based XROMM (X‐ray Reconstruction of Moving Morphology: Brainerd *et al*., 2010). Radiopaque markers were implanted in the bones of the right pelvic limb. After recovery, the subject was induced to run at 1.6 ms^−1^ on a motorized treadmill. Videoradiographs of this activity were taken simultaneously from two angles at 250 Hz (see Kambic *et al*., 2015 for details, all surgical and experimental techniques approved by Brown IACUC).

**Figure 3:**
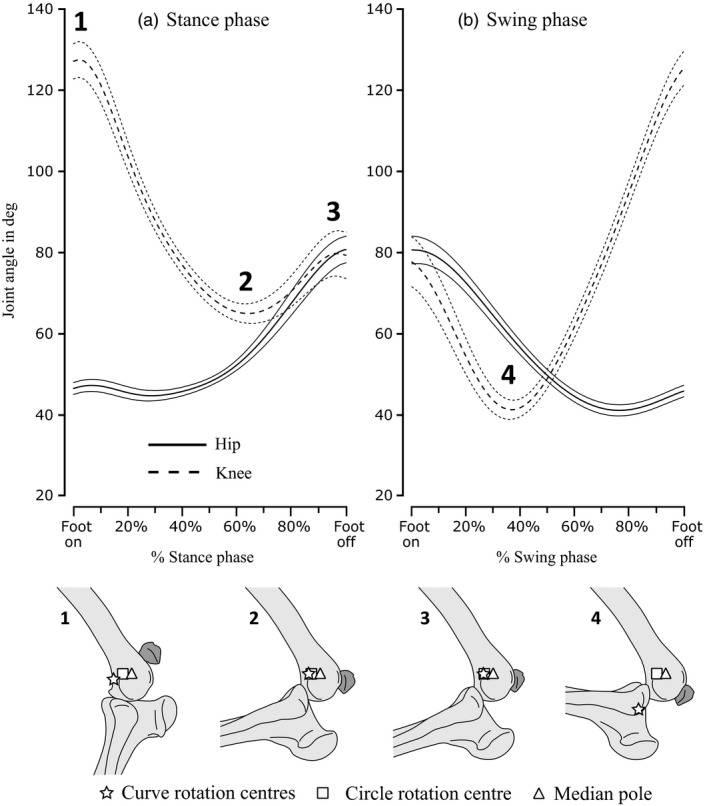
Upper subfigure shows mean joint angles (see Kambic *et al*., 2015) averaged from time‐normalized data from all recorded strides for the hip (solid) and knee (dashed) joints, plotted against percentage stance and swing phases. Values one standard deviation ± from the mean value (thick lines) are shown as thinner lines in the same format. Lower subfigure shows patella position relative to the knee at the minima and maxima of knee angles numbered 1–4 on the upper subfigure, with estimated positions of rotation centres shown for the curve‐fit (start), circle‐fit (square) and median‐pole (triangle) methods.

Markers' positions were digitized from both X‐ray videos, and the resulting 3D coordinates used to reconstruct skeletal kinematics using the XrayProject, a set of freely available (www.xromm.org) Matlab (MathWorks, Natick, MA, USA) and Maya (Autodesk Inc., San Rafael, CA, USA) scripts. Kinematic data were filtered at 15 Hz (Fourth order Butterworth, low‐pass) in Matlab and used to animate a digital model of the subject in Maya. Joint coordinate systems were added to the hip and knee (See Kambic *et al*., 2015 for details), allowing joint angles to be calculated (Fig. 3). Joint angle conventions follow Kambic *et al*. (2015) – hip extension angle is 0° when the femur points cranially, parallel to the wings of the ilium; at a hip angle of 90°, the femur points ventrally. The knee is fully extended at 180°, smaller knee angles are more flexed.

Only a single marker was implanted in the patella, and so its relative position was recorded but not its orientation. Based on manipulation of a cadaver and CT data from an articulated hindlimb, we adjusted patella orientation in Maya so that the patella was either kept in articulation with the patellar groove or had its articular surface facing the femur throughout the stride cycle.

Limb phase was estimated from foot on/off timing taken from synchronized standard light video. Skeletal kinematics were time‐normalized to percentage stance or swing (using cubic spline interpolation in Matlab) to allow comparison between strides. Joint flexion‐extension angles are available in Table S1. Raw X‐ray video (*All Studies/Guineafowl Long Axis Rotation study/GFLAR06RUN70*) and calibration data (*All Studies/Guineafowl Long Axis Rotation study/20130522GFLAR06treadpre*) are publicly available at the X‐ray Motion Analysis Research Portal (xmaportal.org).

### Muscular anatomy and estimation of lines of action

The anatomy of the patellar complex of *Numida* was taken from a diffusible‐iodine contrast‐enhanced micro‐CT scan (e.g. Gignac & Kley, 2014) of a separate individual, which had died for reasons unrelated to this study. Body mass was reasonably close to that of our kinematics specimen (1.65 kg). The specimen was fixed in 10% neutral‐buffered formalin for 3 days, flushed with phosphate‐buffered saline and then immersed in 7.5% I_2_KI in aqueous solution for 19 days. The specimen was then scanned at 0.2 mm resolution with a Nikon XT 225 ST microCT at the Cambridge Biotomography Centre, UK.

Bones and MTU bellies were segmented from micro‐CT data as 3D volumes in AMIRA software (FEI, Waltham, MA, USA). In addition to the patellar tendon, three knee extensor MTU's were found to directly attach to the patella: *M. iliotibialis cranialis* (IC)**,** *M. iliotibialis lateralis preacetabularis* (ILPR) and *M. femorotibialis intermedius* (FTI) (See Fig. 2a for anatomy). From the segmented volumes areas of contact between each MTU belly and the relevant bones (representing anatomical origin and insertion) were extracted in Matlab (code available from FigShare [https://doi.org/10.6084/m9.figshare.4595821]). We then took cross‐sections of each belly volume along an axis running from origin to insertion. The line‐of‐action was then assumed to be a path through the geometric centre of each cross‐section (Fig 2). MTU path markers were then scaled, translated and attached to the bones of our kinematics specimen, allowing their positions for each kinematic frame to be output.

### Muscle activity

Electromyography (EMG) activity for the IC, ILPR and FTI of running *Numida* has been recorded by previous studies (Gatesy, 1999b; Marsh *et al*., 2004). We restricted our analysis to those periods of stance and swing when these MTUs are active, assuming that any passive forces that they generated are negligible.

### Estimation of movement planes

To satisfy the assumption of planarity for the patellar complex, we estimated a plane‐of‐best‐fit for digitized markers on the tibiotarsus and patella using the ‘pca’ principle component analysis function in Matlab. Out‐of‐plane variance in marker position an order of magnitude lower than in‐plane variance was considered to represent satisfactorily planar motion. Skeletal kinematics were transformed to lie in the plane‐of‐best‐fit and subsequently considered to be 2D.

### Estimation of centres of rotation

To give good results, simple methods for estimating instantaneous rotation centres (e.g. Alexander, 1983; Hamill, Selbie & Kepple, 2013) require smooth, planar motion. This makes identification of instantaneous rotation centres for complex biological mechanisms such as the patellofemoral joint difficult. In Alexander & Dimery's (1985) study of *ex vivo* and *in vivo* sesamoid mechanics, they substituted planar motion poles, calculated for endpoints of large angular displacements, for instantaneous centres. This assumes that any intermediate motion was smooth, planar and circular.

Here, we used several methods for estimating rotation centres for the patellofemoral joint. Firstly, we calculated average planar movement poles for our marker positions according to the method of Hamill *et al*. (2013). We could arrive at no good criteria for choosing a single pair or small number of frames from our kinematics (as in Alexander & Dimery, 1985), and so instead calculated an average movement pole from estimates done for every sequential pair of frames. Even with low‐pass filtering, our patellar motion was insufficiently smooth, with estimates of pole location sometimes translating large distances instantaneously between pairs of frames. We therefore calculated a median rather than a mean movement pole, to avoid undue influence of these outliers.

The second and third methods we employed used curves‐of‐best‐fit to smooth our kinematic data. The simpler of the two fitted a circular path to marker positions plotted over time, using the ‘CircleFitByPratt’ function written by N. Chernov for Matlab (https://uk.mathworks.com/matlabcentral/fileexchange/22643-circle-fit-pratt-method). The centre of rotation for all points was then assumed to be at the centre of the fitted circle. For the final method, we fitted a second‐order polynomial curve to our points, using the ‘polyfit’ function in Matlab. The centre of rotation for each point was assumed to be at the instantaneous centre of curvature for the corresponding point on the curve, calculated by projecting the instantaneous radius of curvature along the instantaneous curve normal.

The second‐order polynomial fit allowed the curvature of the path fitted to marker positions to vary, and so the estimated rotation centre moved with the bones, unlike in the previous two methods. To assess whether a circular or variably curved polynomial path was the best fit for our kinematic data, *R* ^2^ values were computed for each. Estimated centres and *R* ^2^ values are shown in Fig. S2.

### Estimation of moment arms

**Figure 4:**
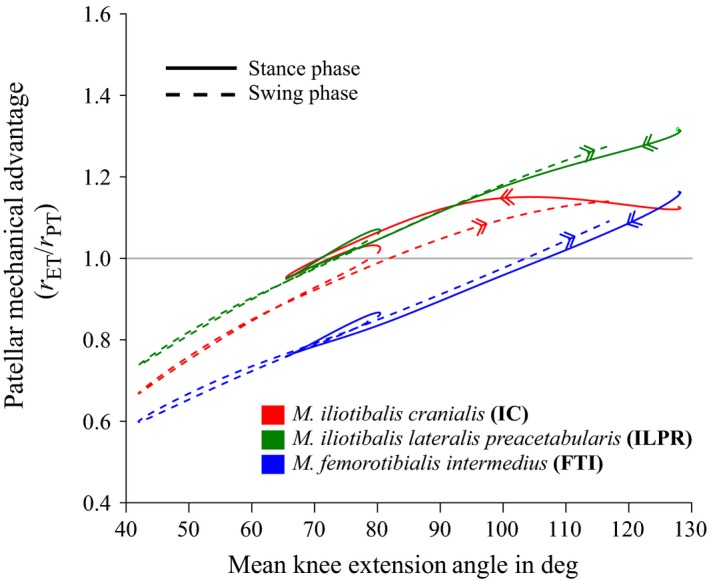
Mean values of *r*_ET_/*r*_PT_, taken to be a proxy for mechanical advantage of the patellofemoral joint (see Materials and methods). Data were averaged from time‐normalized data from all recorded strides and plotted against mean knee extension angle (averaged in the same way), for the muscles *M. iliotibialis cranialis* (red), *M. iliotibialis lateralis preacetabularis* (green) and *M. femorotibialis intermedius* (blue). Data from stance phase are shown as thick, solid lines, and data from swing phase are shown as thinner, dashed lines. Arrows on each curve indicate progression from stance to swing. All data were produced using curve‐fit rotation centres (see Materials and methods).

**Figure 5:**
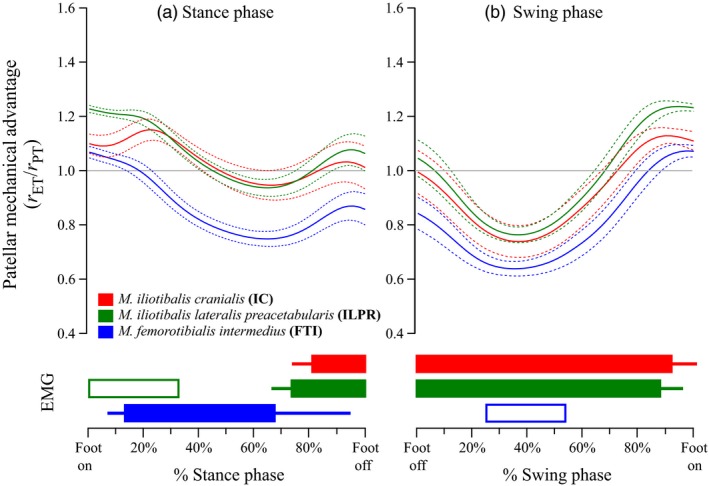
Mean values of *r*_ET_/*r*_PT_, taken to be a proxy for mechanical advantage (see Materials and methods). Data were averaged from time‐normalized data from all recorded strides, plotted against percentage of (a) stance and (b) swing phases, for the *M. iliotibialis cranialis* (red), *M. iliotibialis lateralis preacetabularis* (green) and *M. femorotibialis intermedius* (blue). The mean value is represented by the thicker lines, with the thinner lines of the same colour representing ±1sd. All data were produced using curve‐fit rotation centres (see Materials and methods). Coloured bars below the main figure show estimated muscle activity over the same time‐normalized stance and swing phases. Filled bars are a consensus between Gatesy (1999b) and Marsh *et al*. (2004), whereas unfilled bars are from Marsh *et al*. (2004) only.

Finally, basic trigonometry was used to calculate moment arms as the perpendicular distance from each MTU path segment to the estimated rotation centre for each frame, measured in our plane‐of‐best‐fit. Three sets of moment arm data were therefore generated, one for each method used to estimate rotation centres. Moment arms for the knee extensor muscles (*r*_ET_) were divided by those for the patellar tendon (*r*_PT_). Means and standard deviations were calculated separately for stance phase and swing phase. Values of *r*_ET_/*r*_PT_ are expressed both relative to knee flexion/extension angle (Fig. 4) and to percentage phase (stance or swing) (Fig. 5). Raw data are available as supplementary information (Table S2).

## Results

Hip and knee kinematics (Fig. 3) matched previous analyses of running guineafowl (Gatesy, 1999a; Rubenson & Marsh, 2009). The hip remained relatively static at ~45° of extension until ~40% into the stance phase (Fig. 3a), after which it extended continuously, reaching ~85° at foot off. This pattern was mirrored in swing, with consistent flexion from ~85° to ~45° at ~60% of swing phase (Fig. 3b). The knee showed a consistent flexion‐extension pattern in both stance and swing, flexing from ~130° to ~65° from 0% to around 60% of stance, then extending to ~80° in late stance (roughly 95%) before beginning to flex again in latest stance (Fig. 3a). In swing, the knee flexed from ~75° to ~45° between 0% and roughly 35% of swing, before rapidly extending to ~130° again in latest swing phase (Fig. 3b).

The three methods used to estimate rotation centres for the patellofemoral joint produced very similar estimates of *r*_ET_/*r*_PT_ (our proxy for mechanical advantage), and so only those for the curve‐fit rotation centres are discussed here, as R^2^ value for the curve‐fit was marginally higher than the circle‐fit (0.767 vs. 0.749; see Figs [Link], [Link] and S4). In both stance and swing, *r*_ET_/*r*_PT_ was highest for the ILPR muscle and lowest for the FTI muscle (Figs 4 and 5). Values of *r*_ET_/*r*_PT_ for all muscles were less than 1.0 at high knee flexion angles and increased with extension (Fig. 4). The angle of knee extension at which *r*_ET_/*r*_PT_ became larger than 1.0 differed for each muscle. For the ILPR it occurred at around 70° of flexion, the IC at around 80 degrees and the FTI at around 105°. There were some differences in values of *r*_ET_/*r*_PT_ at the same angles in stance and swing (Fig. 4, dotted vs. solid lines). In particular, values for the IC were generally higher (by around 0.1) in stance than in swing.

For the first ~20% of the stance phase (Fig. 5a), estimated mechanical advantage (*r*_ET_/*r*_PT_) for all muscles was greater than 1.0 (i.e. force‐enhancing). Subsequently, *r*_ET_/*r*_PT_ values for the FTI dropped below 1.0 (i.e. velocity‐enhancing), reaching a minimum of around 0.8 by 60% of stance. Values for the IC and ILPR declined steadily but remained above 1.0 for the first 50% of stance, before dipping to ~0.9 at ~60% stance. In late stance, values for all three muscles increased slightly – the FTI to around 0.85, the IC to around 1.0 and the ILPR to around 1.05 (Fig. 5a). During the swing phase (Fig. 5b), *r*_ET_/*r*_PT_ values for all muscles were very similar. All declined rapidly between foot off and mid‐swing, reaching a low of around 0.75–0.8 by ~40% of swing. In mid‐late swing, values for all muscles increased, becoming greater than 1.0 by ~70% of swing and peaking at ~1.2 close to foot on.

**Figure 6:**
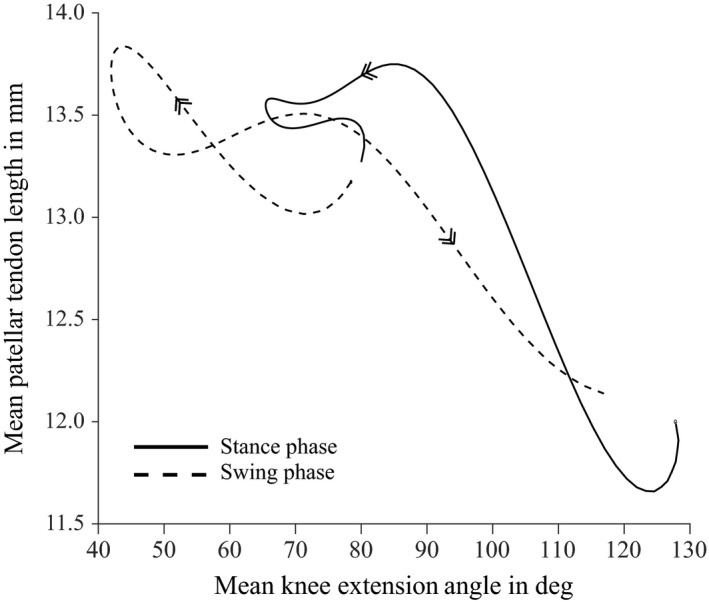
Mean values of patellar tendon length (averaged from time‐normalized data from all recorded strides) plotted against mean knee extension angle (averaged in the same way). Data from stance phases are shown as thick, solid lines, and data from swing phase are shown as thinner, dashed lines. Arrows on each curve indicate progression from stance to swing.

The relationship between knee joint angle and the length of the patellar tendon was found to be inconsistent (Fig. 6). The tendon was up to 0.5 mm longer in stance phase than in swing (mean patellar tendon length was 13.1 mm).

## Discussion

We discuss patellar gearing during the stance phase, followed by the swing phase. EMG activity (shown in the lower part of Fig. 5) indicates that one or more of the FTI, IC and ILPR muscles are active throughout most of the stance. During these periods of activity, our estimates of mechanical advantage (*r*_ET_/*r*_PT_) for all muscles are consistently higher than those reported for humans (Fig. 1 vs. Fig. 5), and at the beginning and end of both stance and swing phase they are greater than 1.0 (typically around 1.1; Fig. 5). This suggests that unlike human patellae, which appear to exclusively enhance knee extension velocity (at the expense of force), during the stance phase of running (Ellis *et al*., 1980; Eijden *et al*., 1986; Biewener *et al*., 2004; data shown in Fig. 1 of this study), the patellae of running *Numida* have a more variable effect on the mechanics of knee extension.

The detailed relationship between estimated patellar gearing, muscle activity and kinematics has interesting implications for how knee movement is actuated, and how muscular and non‐muscular moments are balanced. In the early and middle stance phase, *Numida* (as other birds) retract their limb using mainly knee flexion (Fig. 3a). The many antagonisms and synergisms involved in avian knee dynamics make assignment of functions to individual muscles difficult. However, EMG data suggest that flexion is actuated by the *M. iliofibularis* (IF) and flexor cruris group muscles (‘hamstrings’ equivalents [Gatesy, 1999a,b; Marsh *et al*., 2004]) with assistance from the non‐muscular components of the ground reaction force (GRF), which also exerts a knee flexor moment (Clark & Alexander, 1975).

EMG activity in the FTI and (in early swing) the ILPR (Marsh *et al*., 2004) indicates that these muscles exert simultaneous knee extensor moments as the knee is flexing (Figs 3a and 5a). These antagonistic extensor moments probably prevent limb collapse by over‐flexion (Gatesy, 1999a,b). Our estimates of mechanical advantage are unambiguously greater than 1.0 (force‐enhancement) for the ILPR during its approximate period of early stance activity, but are predominantly below 1.0 (velocity‐enhancement) for the FTI when it is active (Fig. 5a).

Mechanical advantage greater than 1.0 would assist the ILPR in generating supportive (anti‐gravity) moments as the knee flexes. As this mechanical advantage also implies lower output than input velocity, it will also result in greater distal displacement of the ILPR's attachment to the patella. For a monoarticular muscle like the FTI, this requirement for greater displacement can only be met by lengthening the whole MTU. As the ILPR is biarticular, crossing both the hip and knee joints, the displacement requirement may be met at least partially by flexing both the hip and knee (as we see over this period; Fig. 3a), and so might involve less MTU lengthening. Mechanical advantage for the monoarticular FTI is less than 1.0 in early stance, and so (as the output/input velocity ratio is high) the rate of FTI lengthening induced by knee flexion is reduced. Differential patellar gearing in early stance may therefore assist the ILPR in creating large supportive moments about the knee, and also prevent the FTI from being over‐strained and thereby damaged while doing the same via active lengthening.

In middle to late stance, ‘hamstring’ EMG activity ceases, while that of the FTI continues (Gatesy, 1999b; Marsh *et al*., 2004), consistent with slowing knee flexion and initiating knee extension by around 70% stance (Fig. 3a). Estimated mechanical advantage for the FTI was consistently below 1.0 and declined steadily over this period, reaching a minimum of ~0.75 at 70% stance, approximately the same time knee extension begins (Fig. 5a). Patellar gearing may consequently be increasing the force required from the FTI to support the limb against gravity (i.e. the GRF).

Knee flexion resumed in late stance and continued into early swing (Fig. 3), protracting the limb in combination with hip flexion. EMG data show that the biarticular IF is active at this time in guineafowl, exerting both hip extension and knee flexion moments, and also the IC and ILPR, exerting hip flexion and knee extension moments (Gatesy, 1999b; Marsh *et al*., 2004; Fig. 5). Actuating both hip and knee flexion while avoiding limb collapse therefore involves careful balance of antagonistic moments, and is further complicated by the GRF, which imposes an additional flexor moment in latest stance (when knee flexion is initiated) but is absent in swing (when knee flexion continues). We estimate mechanical advantage to be greater than 1.0 for the ILPR (and with less confidence, the IC) in late stance, and to rapidly decline to less than 1.0 for both by ~10% swing (Fig. 5b). This pattern would alter relative extensor moment magnitudes in a similar pattern to that expected for flexor moments as the GRF is removed.

In mid‐to‐late swing (from ~40% onwards, Fig. 3b), rapid knee extension started. EMG data indicate this is actuated by the IC, ILPR and (briefly) the FTI (Gatesy, 1999b; Marsh *et al*., 2004; Fig. 5b). Our estimates of mechanical advantage for mid‐swing were lower than 1.0 for all muscles (~0.8 at ~40% swing, Fig. 5b). As this implies higher output velocity, patellar gearing may also assist the actuation of rapid knee extension in mid‐late swing.

Although the knee joint moved through the same angles multiple times during the gait cycle (Fig. 3a), the relationship between joint angle and estimated mechanical advantage was somewhat inconsistent (Fig. 4). For the ILPR and particularly the IC, *r*_ET_/*r*_PT_ was lower in swing than in stance, whereas for the FTI values were lower in stance than swing (Fig. 4, dashed vs. solid lines). We attribute this inconsistency to variable patellar tendon strain. The estimated magnitudes of *r*_ET_ and *r*_PT_ were determined by the relative positions of the femur, tibiotarsus and patella (Fig. 2). Although knee angle determines femur and tibiotarsus position, patellar position also depends on the length of the patellar tendon, which represents the distance from the cnemial crest to the patella (Fig. 2b). If the patellar tendon does not change length, then a consistent relationship is expected between knee joint angle and patellar position, and so also *r*_ET_/*r*_PT_. However, our analysis implied that the patellar tendon changed length by around 1.5 mm as it was subjected to variable forces during the gait cycle (Fig. 6), and so we saw a variable relationship between knee joint angle and estimates of *r*_ET_/*r*_PT_.

Equivalent data from other species are rare, as most studies of pelvic limb moment arms in species with patellae either ignore it (e.g. Smith *et al*., 2007; Johnson *et al*., 2008; Charles *et al*., 2016) or do not analyse the patellofemoral joint separately from the knee joint (e.g. O'Neill *et al*., 2013). Alexander & Dimery (1985) report mean patellar mechanical advantage of 0.75 for a camel (*Camelus dromedarius*), whereas Regnault *et al*. (in press) report values of 0.66–0.80 for an ostrich (*Struthio camelus*). Our results therefore indicate generally higher patellar mechanical advantage for *Numida* than for other species studied, including humans (Fig. 1 vs. Fig. 5). The disparity in anatomy and kinematics between these species (as well as the methods used to study them) makes meaningful comparisons difficult, but *Numida* is the smallest by several orders of magnitude. Small animals use generally more crouched limbs, prolonging the stance phase and potentially avoiding costs associated with rapid generation of force when running quickly (Kram & Taylor, 1990), at the cost of reduced overall limb mechanical advantage (Biewener, 1989). Higher patellofemoral mechanical advantage in *Numida* versus larger animals may help counteract this decrease at the knee.

However, our findings imply that variable patellar tendon length can affect estimates of mechanical advantage (Fig. 6). It is worth noting that much of the comparative data are taken from *ex vivo* studies in which a static force was applied artificially to the knee extensor tendon (Bishop, 1977; Ellis *et al*., 1980; Eijden *et al*., 1986; Regnault *et al*., in press). Such *ex vivo* studies may not fully account for the effects of dynamic patellar tendon strain under *in vivo* muscle forces, which could explain some of the differences observed between our (*in vivo*) dataset and others.

However, the validity of our results depends on the accuracy of our assumptions about the planarity, lack of friction and rotation centre location for the patellofemoral joint, as well as the accuracy of our estimations of muscle lines of action. A modified *ex vivo* experiment, recording tendon tensions from the patellar complex of an instrumented *Numida* cadaver(s) subjected to dynamic extensor forces estimated from *in vivo* data, would perhaps represent the best of both methods, or at least a useful test of the methods and data presented here. However, estimation of the individual muscle forces needed for such an experiment requires a musculoskeletal simulation based on complete skeletal kinematics, limb endpoint forces, segment inertial properties and detailed muscle–tendon unit properties (see Hutchinson *et al*., 2015 for an equivalent model of an ostrich). As such, it is beyond the scope of this project, but is a promising line of enquiry for future work. One obvious caveat is that the model would also require accurate estimates of joint centres and muscle lines of action, which if estimated using a similar method to ours here, carries a risk of circularity that may reduce the value of such a model to validation experiments.

Finally, as our results indicate similar patellar gearing effects in humans and *Numida* (two disparate bipeds) as well as camels and ostrich, they strengthen the hypothesis that knee extensor muscle sesamoids in general act as gears. Unfortunately, studies of patellofemoral mechanics in birds, lizards or (non‐human) mammals are rare, and thus far mostly involve only one representative individual (as we do here). The extent of inter‐individual versus inter‐species variation in patellar gearing is therefore unknown, and so more studies (particularly on lizards) involving more individuals are needed before both this and alternate hypotheses can be tested with confidence. A promising line of enquiry may stem from recent work inferring that patellae in birds (Regnault *et al*., 2014), lizards (Regnault *et al*., 2016) and mammals (Samuels, Regnault & Hutchinson, 2017) are unevenly phylogenetically distributed, even among quite closely related taxa. Direct comparison between the knee mechanics of closely related patella‐bearing and patella‐lacking species would be a logical next step in developing our understanding of the roles these sesamoid bones play in locomotion.

## Supporting information


**Figure S1**. Mean knee joint flexion/extension angles for specimens of *Numida* running at various speeds (see Kambic *et al*., 2015) averaged from time‐normalized data from all recorded strides, plotted against percentage stance and swing phases.

**Figure S2.** Estimated rotation centres for the patellofemoral joint, shown in the plane‐of‐best‐fit for patella motion.

**Figure S3.** Mean values of *r*_ET_/*r*_PT_, taken to be a proxy for mechanical advantage (see Materials and methods).

**Figure S4.** Mean values of *r*_ET_/*r*_PT_, taken to be a proxy for mechanical advantage (see Materials and methods).

**Table S1.** Flexion‐extension angles in degrees for the hip and knee joints, with numbered limb phases. See S*keletal Kinematics* section of Materials and methods for details.

**Table S2.** Moment arms calculated for *M. iliotibialis cranialis* (IC), *M. iliotibialis lateralis preacetabularis* (ILPR) and *M. femorotibialis intermedius* (FT) about patellofemoral rotation centres estimated by second‐order curve fitting (Curve Centres), circular arc fitting (Circle Centre) or median planar movement poles (Median Pole), with numbered limb phases.
