# Supplementary figures and images for: Gearing effects of the patella (knee extensor muscle sesamoid) of the helmeted guineafowl during terrestrial locomotion
Source: J Zool (1987). 2017 Jul 19;303(3):178–87. doi: 10.1111/jzo.12485 (PMC5697681; doi:10.1111/jzo.12485)

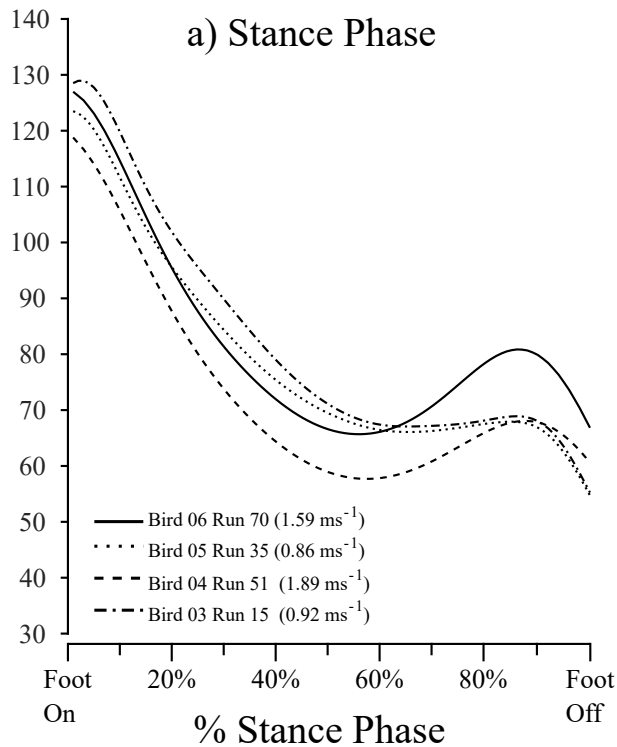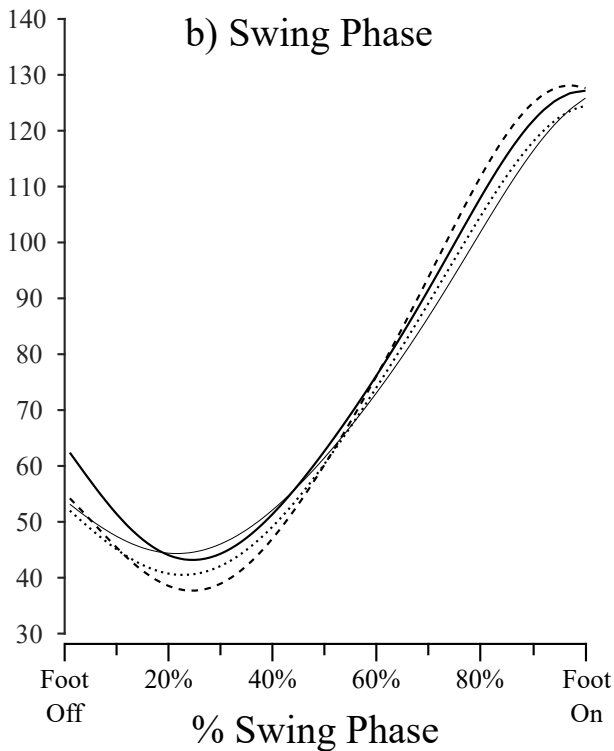

Supplement: Supplementary file 1 — Figure S1. Mean knee joint flexion/extension angles for specimens of Numida running at various speeds (see Kambic et al., 2015) averaged from time‐normalized data from all recorded strides, plotted against percentage stance and swing phases. [file JZO-303-178-s001.pdf]

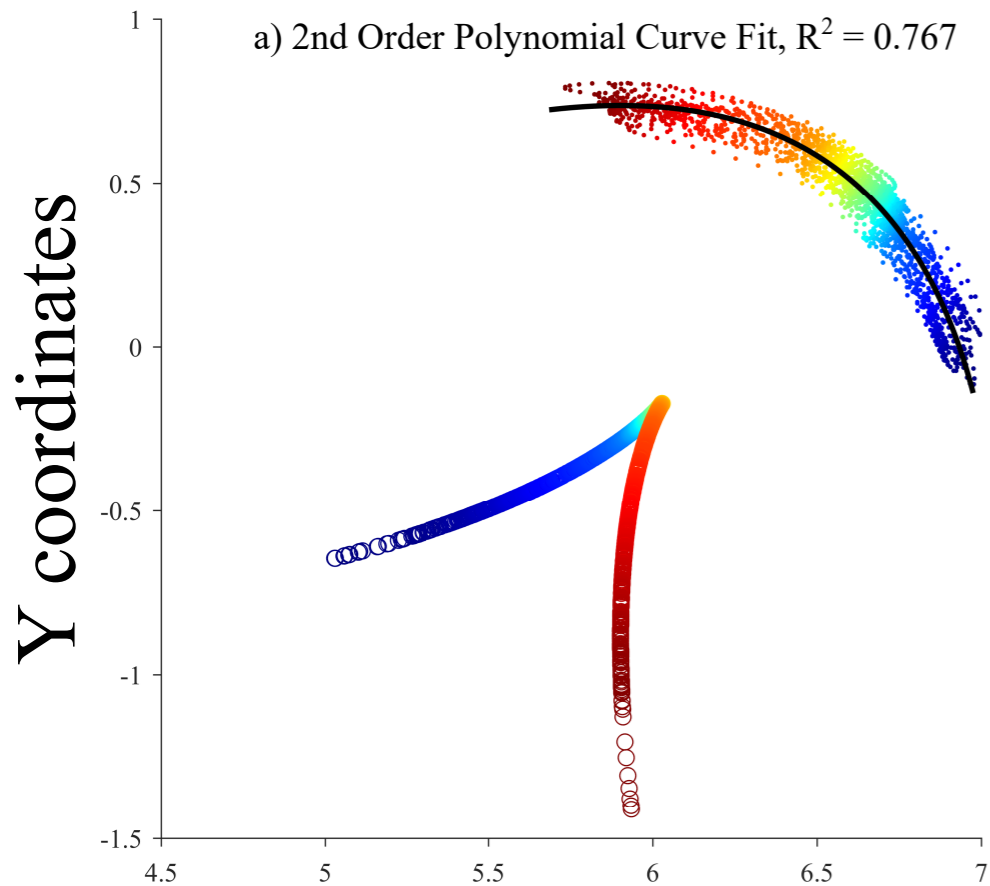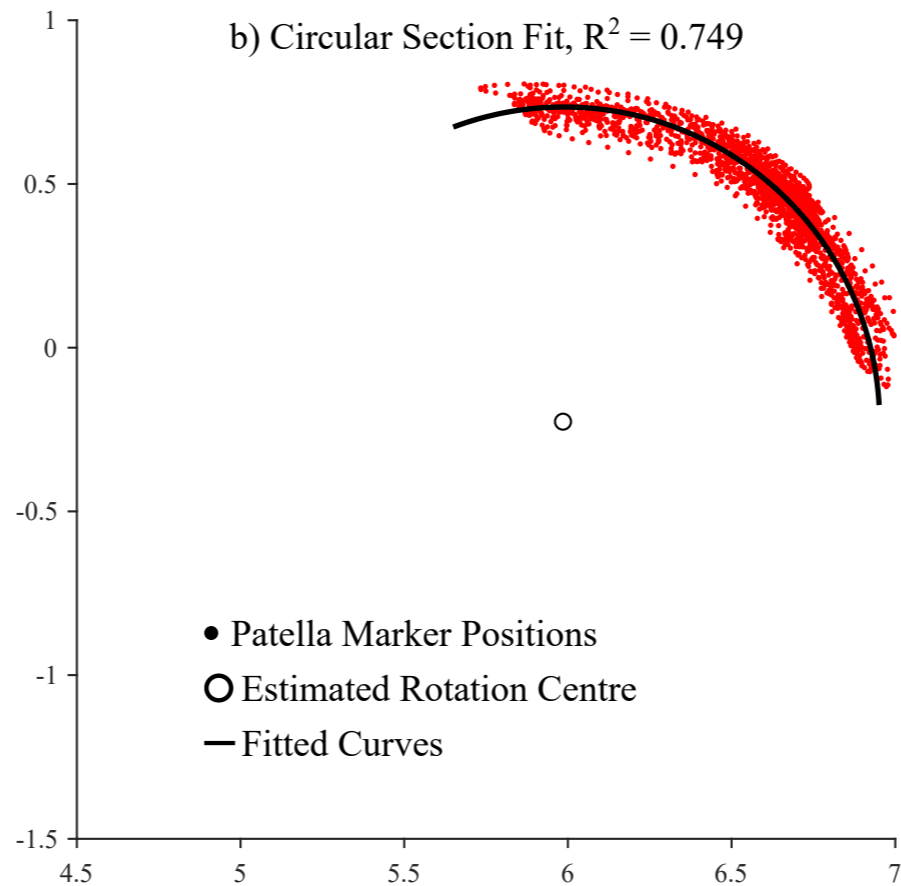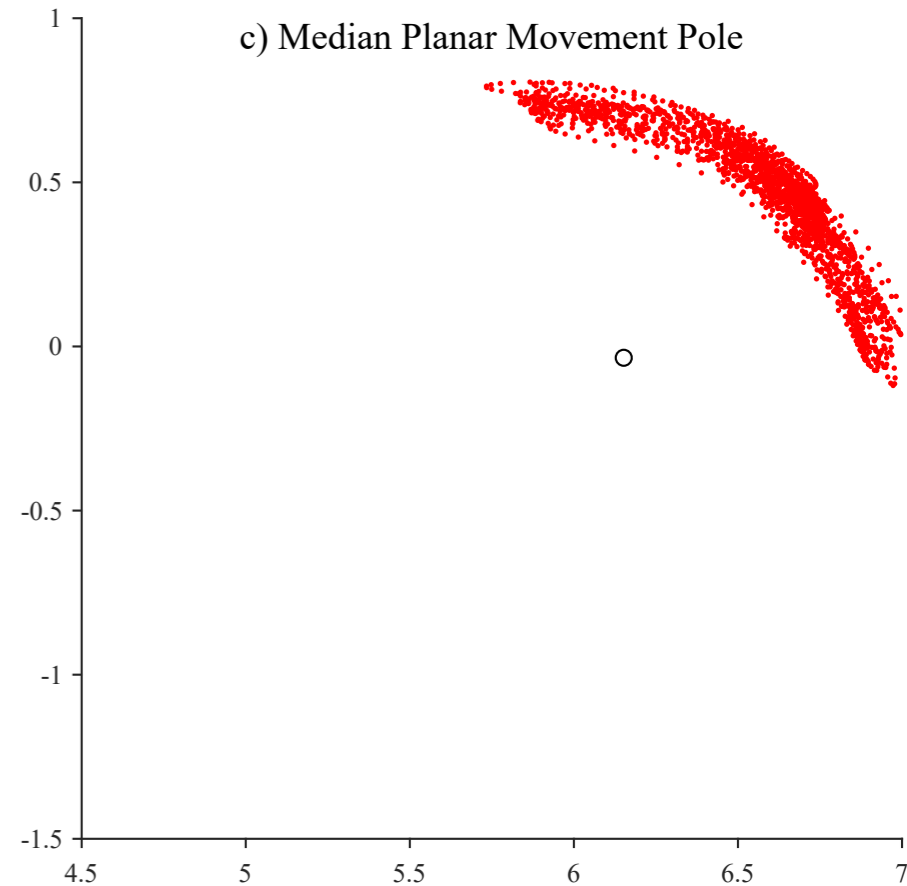

Supplement: Supplementary file 2 — Figure S2. Estimated rotation centres for the patellofemoral joint, shown in the plane‐of‐best‐fit for patella motion. [file JZO-303-178-s002.pdf]

a) Curve Rotation Centres

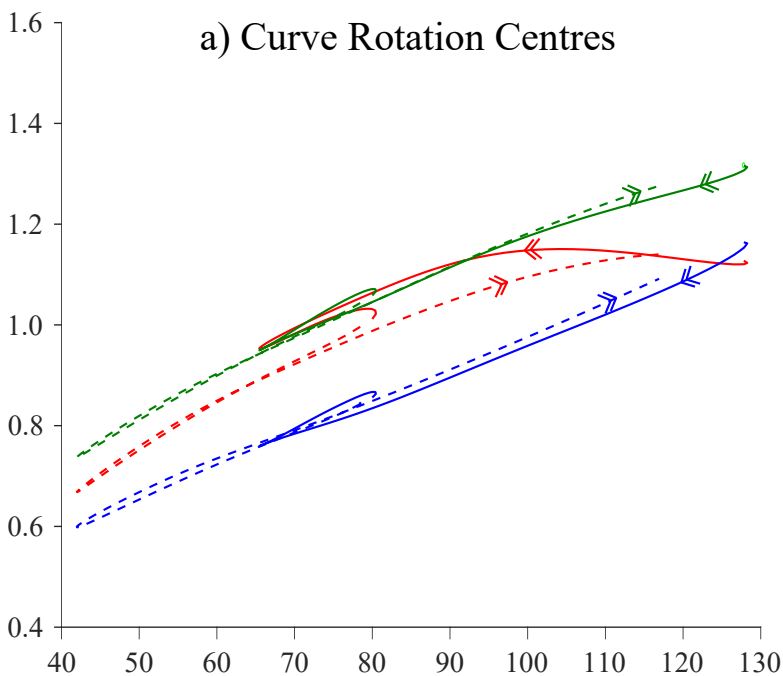

b) Circle Rotation Centre

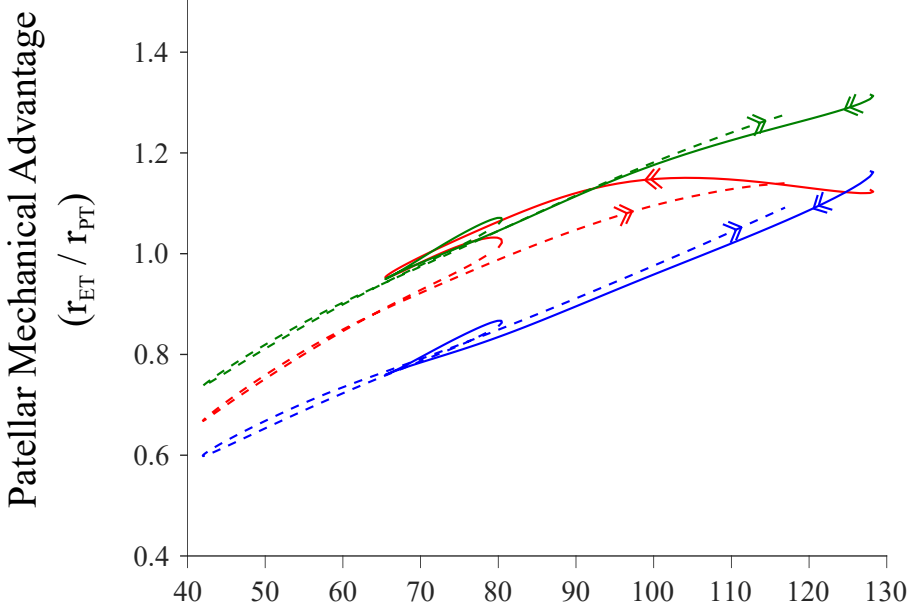

c) Median Pole

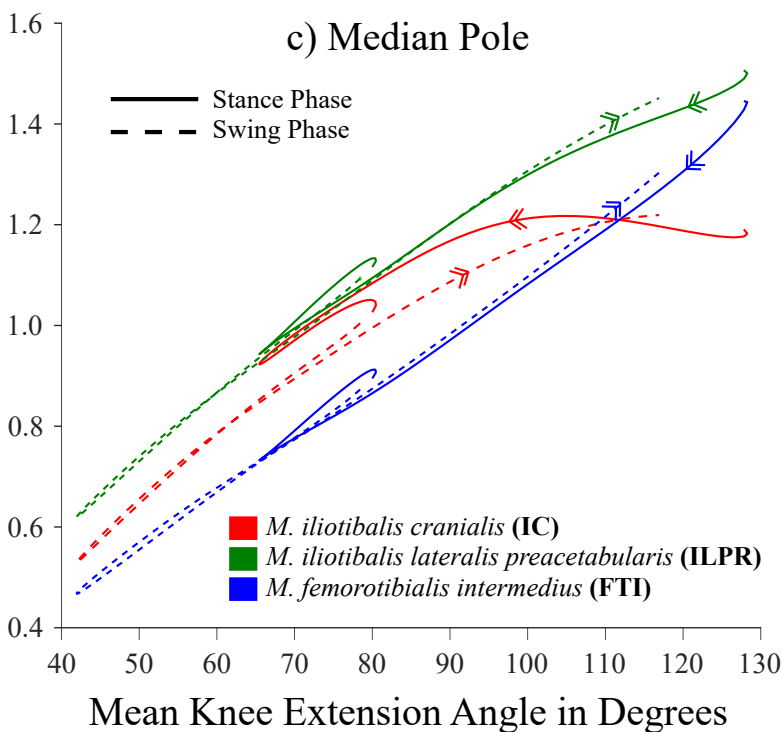

Supplement: Supplementary file 3 — Figure S3. Mean values of r ET/r PT, taken to be a proxy for mechanical advantage (see Materials and methods). [file JZO-303-178-s003.pdf]

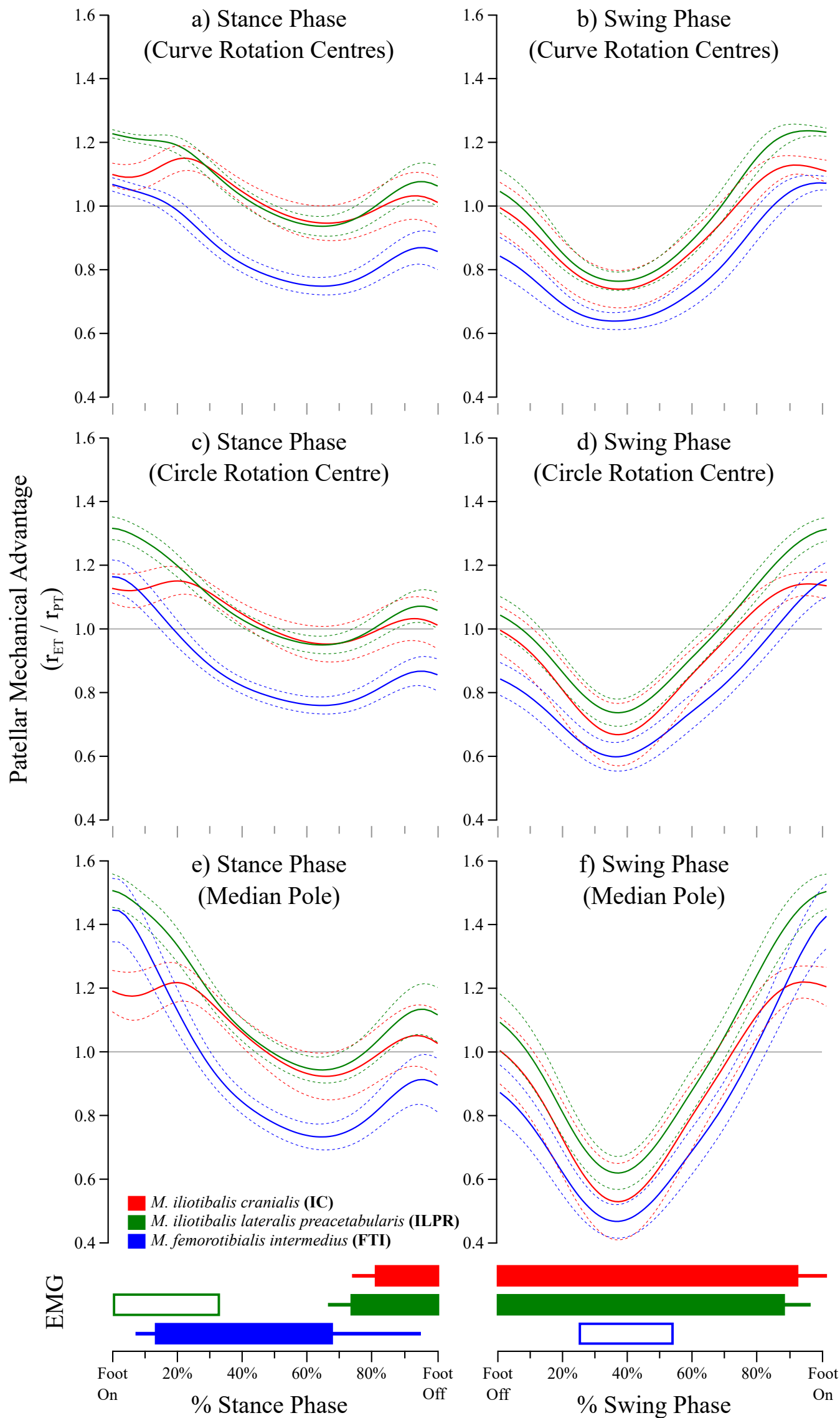

Supplement: Supplementary file 4 — Figure S4. Mean values of r ET/r PT, taken to be a proxy for mechanical advantage (see Materials and methods). [file JZO-303-178-s004.pdf]
