# Supplementary material for: Gearing effects of the patella (knee extensor muscle sesamoid) of the helmeted guineafowl during terrestrial locomotion
Source: J Zool (1987). 2017 Jul 19;303(3):178–87. doi: 10.1111/jzo.12485 (PMC5697681; doi:10.1111/jzo.12485)
Supplement: Supplementary file 7 [file JZO-303-178-s007.docx]

**SUPPLEMENTARY INFORMATION LEGENDS:**

**SUPPLEMENTARY TABLES**

**Table S1:** Flexion-extension angles in degrees for the hip and knee joints, with numbered limb phases. See S*keletal Kinematics* section of Methods for details.

**Table S2:** Moment arms calculated for *M. iliotibialis cranialis* (IC), *M. iliotibialis lateralis preacetabularis* (ILPR) and *M. femorotibialis intermedius* (FT) about patellofemoral rotation centres estimated by 2^nd^ order curve fitting (Curve Centres), circular arc fitting (Circle Centre) or median planar movement poles (Median Pole), with numbered limb phases. See *Estimation of centres of rotation* and *Estimation of moment arms* sections of Methods, and supplementary figure S2 for details.

**SUPPLEMENTARY FIGURES**

**Figure S1**: Mean knee joint flexion/extension angles for specimens of *Numida* running at various speeds (see Kambic et al. 2015) averaged from time-normalised data from all recorded strides, plotted against percentage stance and swing phases. Specimen number, run number and speed are indicated in the legend. Raw X-ray videos are publically available at the X-ray Motion Analysis Research Portal (xmaportal.org). All trials are located in *All Studies/ Guineafowl Long Axis Rotation study* under subheadings *GFLAR06RUN70* (Bird 6, Run 70), *GFLAR05RUN35* (Bird 5, Run 35), *GFLAR04RUN51* (Bird 4, Run 51) and *GFLAR03RUN70* (Bird 3, Run 15).

**Figure S2:** Estimated rotation centres for the patellofemoral joint, shown in the plane-of-best-fit for patella motion. Dots indicate patella marker positions, hollow circles estimated rotation centre(s), and lines are curves fitted to the data. Shown in **a)** are rotation centres estimated as the centre of curvature for a 2^nd^ order polynomial fitted to patella marker data. Marker data and estimated centres were colour-coded to facilitate legibility – blue markers relate to blue rotation centres, red to red, and so forth. Shown in **b)** is the rotation centre produced from the centre of curvature of a circular arc fitted to patella marker data, and shown in **c)** is the median planar movement pole, calculated from planar movement poles taken from each sequential pair of marker positions.

**Figure S3:** Mean values of r_ET_ / r_PT_, taken to be a proxy for mechanical advantage (see Methods). Data were averaged from time-normalised data from all recorded strides and plotted against mean knee extension angle (averaged in the same way), for the *M. iliotibialis cranialis* (red), *M. iliotibialis lateralis preacetabularis* (green) and *M. femorotibialis intermedius* (blue). Data from stance phase are shown as thick, solid lines, and data from swing phase are shown as thinner, dashed lines. Arrows on each curve indicate progression from stance to swing. Data were produced in **a)** using patellofemoral rotation centres estimated by 2^nd^ order curve fitting (Curve Rotation Centres), **b)** using circular arc fitting (Circle Rotation Centre) and **c)** using median planar movement poles (Median Pole). See *Estimation of centres of rotation* and *Estimation of moment arms* sections of Methods, and supplementary figure S2 for details.

**Figure S4:** Mean values of r_ET_ / r_PT_, taken to be a proxy for mechanical advantage (see Methods). Data were averaged from time-normalised data from all recorded strides and plotted against plotted against percentage of stance (left column) and swing (right column) phase, for the *M. iliotibialis cranialis* (red), *M. iliotibialis lateralis preacetabularis* (green) and *M. femorotibialis intermedius* (blue). Data in **a)** and **b)** were produced using patellofemoral rotation centres estimated by 2^nd^ order curve fitting (Curve Rotation Centres), **c)** and **d)** using circular arc fitting (Circle Rotation Centre) and **e)** and **f)** using median planar movement poles (Median Pole). See *Estimation of centres of rotation* and *Estimation of moment arms* sections of Methods, and supplementary figure S2 for details.
